# Benchmark dataset for the Voronoi diagram of 3D spherical balls

**DOI:** 10.1016/j.dib.2022.108605

**Published:** 2022-09-14

**Authors:** Chanyoung Song, Mokwon Lee, Seunghwan Choi, Deok-Soo Kim

**Affiliations:** a School of Mechanical Engineering, Hanyang University, 222 Wangsimni-ro, Seongdong-gu, Seoul, South Korea; b Voronoi Diagram Research Center, Hanyang University, 222, Wangsimni-ro, Seongdong-gu, Seoul, South Korea

**Keywords:** Sphere, Coordinate, Random, Tessellation, Computational geometry

## Abstract

In this paper, we present a dataset to be used for the construction of the Voronoi diagram of 3D spherical balls (VD-B3). The dataset consists of sphere arrangements including general, anomaly, and extreme cases. The dataset also includes protein models downloaded from RCSB Protein Data Bank (PDB). The dataset can be used as a standard benchmark dataset to verify and validate the correctness, efficiency, and robustness of the construction algorithm. The dataset is simple and easy to understand. The details of the experiment and analysis based on this dataset are presented in the original research article: “Robust Construction of the Voronoi Diagram of Spherical Balls in the Three-Dimensional Space” which introduces the topology-oriented incremental algorithm for the construction that is thoroughly validated and compared with two implementations of the well known edge-tracing algorithm.

## Specifications Table

**Table:** 

Subject	Geometry and Topology
Specific subject area	Computational geometry
Type of data	1) Text files (Each file represents an arrangement of 3D spherical balls) 2) PDB files (Each file represents a protein model that consists of 3D spherical balls)
How the data were acquired	1) Text files are generated by a C++ program on Intel® Core™ i7-7700 3.60GHz, 16.GB RAM with Window 10 operating system. 2) PDB files are downloaded from RCSB Protein Data Bank.
Data format	1) Text files: Raw data 2) PDB files: Curated data
Description of data collection	• Sets of random 3D spherical balls within a spherical container: BALLCLOUD, BALLSMALLSET[1,10], VISUALSET (Vis-I-10). Hereafter a ball denotes a 3D spherical one. • Sets of random balls touching a reference sphere: EXTREMESET, VISUALSET (Vis-V-20, Vis-VI-6, Vis-VII-20) • A set of balls on a grid: VISUALSET (Vis-IV-60) • Sets of balls for anomaly case in Voronoi diagram: ANOMALYSET, VISUALSET (Vis-II-5, Vis-III-5) • A set of protein models downloaded from RCSB Protein Data Bank: PROTEINSET
Data source location	1) Text files: Voronoi Diagram Research Center, Hanyang University, Seoul, South Korea 2) PDB files: RCSB Protein Data Bank
Data accessibility	Repository name: Benchmark Dataset for the Voronoi Diagram of 3D Spherical Balls Direct URL to data: https://data.mendeley.com/datasets/jgb8g5m6k9 DOI: 10.17632/jgb8g5m6k9.1
Related research article	M. Lee, K. Sugihara, D.-S. Kim, Robust Construction of Voronoi Diagrams of Spherical Balls in the Three-Dimensional Space, Comput.-Aided Des. 152 (2022) 103374. https://doi.org/10.1016/j.cad.2022.103374

## Value of the Data

• The Voronoi diagram of 3D spherical balls (VD-B3) include the analysis and the design of biomolecular structures, e.g., proteins and material structures. The predictions of collisions between drones, airplanes and satellites are also emerging applications. Despite of its importance for solving such diverse applications, the robust construction of VD-B3 is an extreme challenge and has long been a hot research topic. A standard benchmark dataset is therefore desirable or necessary for researchers.
• The dataset contains general cases (without an anomaly), anomaly cases, and extreme cases as well as protein models for validating the correctness, efficiency, and robustness of the algorithm to construct VD-B3.
• Researchers who develop and/or evaluate the algorithm of VD-B3 can benefit from the dataset.
• The dataset can be used as a benchmark dataset to compare the performance of VD-B3 algorithms.

## 1 Data Description

Here, we present a dataset to be used for the construction of the Voronoi diagram of 3D spherical balls (VD-B3). The dataset consists of six types of file collection: BALLCLOUD, BALLSMALLSET[1,10], EXTREMESET, ANOMALYSET, PROTEINSET, and VISUALSET. VISUALSET is for the visual check of constrcuted Voronoi diagrams and the others are for computational experiments. PROTEINSET consists of PDB files describing the 3D structures of biomolecules. Except those in PROTEINSET, balls do not intersect each other. The details of experiments and analysis using this dataset can be found in the original research article [1]. This dataset can be downloaded from our Mendeley Data repository [2]. Hereafter, a ball denotes a 3D spherical one.

1) BALLCLOUD

BALLCLOUD = {BALL[1,1], BALL[1,2], BALL[1,5], BALL[1,10]} consists of four types of sphere arrangements where BALL[1,i] = {Ball_1_[1,i], Ball_2_[1,i], …, Ball_10_[1,i]} is a set of ten files of random balls whose radii are randomly chosen in [1.0, i] (1.0 ≤ i). Each Ball_j_[1,i] has 10,000*j balls in itself. Each file Ball_j_[1,i] is named as ‘BALL_1_i_j0000.txt’ and stored in ‘BALLCLOUD\BALL_1_i’ folder of the repository.

2) BALLSMALLSET[1,10]

BALLSMALLSET[1,10] = {Ball_1_^small^, Ball_2_^small^, …, Ball_30_^small^} consists of 30 files of random balls where Ball_j_^small^ has 1,000 * j balls whose radii are from [1, 10]. Each file Ball_j_^small^ is named as ‘BALL_SMALL_j000.txt’ and stored in ‘BALLSMALLSET’ folder of the repository.

3) EXTREMESET

EXTREMESET = {Ext-I-Congruent-300, Ext-II-Polysized-300} consists of two files with 300 balls tangent to a reference sphere, respectively. Both files are to test the extreme cases of degeneracy or near degeneracy. Ext-I-Congruent-300 has identical-sized balls (radius: 1) while Ext-II-Polysized-300 has poly-sized balls (radius: [1,2]). The radius of the reference sphere is fixed as 80. Both are in ‘EXTREMESET’ folder of the repository.

4) ANOMALYSET

ANOMALYSET = {ANO1, ANO2, ANO3, ANO4} is a set of four files which contains anomaly cases in VD-B3. In ANO1, an arrangement of two large balls and a small ball between the two large ones exists to define an elliptic Voronoi edge (V-edge) e which has no Voronoi vertex (V-vertex). As a consequence, e is not connected to any V-edge in the big-world. In this sense, this case is called “0-connected". The e constitutes a small-world on its own. In ANO2, ANO1 case occurs twice in a nested fashion. In ANO3, there are three tiny balls between two large balls. Their arrangement forms a small-world which consists of four V-vertices. This case is called “3-connected" because two V-vertices among the four ones are connected with three V-edges, if they are. In ANO4, the two V-vertices in the small-world are connected to each other with all four available V-edges. Hence, this case is called “4-connected". For details of anomalies, see [3]. Each file is stored in ‘ANOMALYSET’ folder of the repository.

5) PROTEINSET

PROTEINSET consists of 20 protein models downloaded from RCSB PDB [4] where the protein models consist of atomic coordinates calculated using the Fourier transformation of electron density maps of a protein crystal [5]. Table 1 shows the 20 protein models and the number of atoms which constitute the models.

**Table 1:** 20 protein models in PROTEINSET

PDB code	#atoms	PDB Code	#atoms	PDB Code	#atoms	PDB Code	#atoms
1j3h	4,924	4av7	29,640	4hel	54,096	6d5f	79,946
5cqs	9,786	3re7	34,032	4a13	60,012	6fai	84,916
5ljv	14,832	6eos	39,873	4rkm	62,698	3jaq	89,373
6dbi	19,995	6f42	45,039	3opy	64,576	1o1a	91,996
3uq7	25,000	5l5z	49,356	3ias	74,632	5vfr	99,783

6) VISUALSET

VISUALSET = {Vis-I-10, Vis-II-5, Vis-III-5, Vis-IV-60, Vis-V-20, Vis-VI-6, Vis-VII-20} consists of seven files for visual inspection purpose. Vis-I-10 has 10 random balls with an identical radius. Vis-II-5 and Vis-III-5 are data to make anomaly cases with nested small worlds and with stack-up small worlds in VD-B3, respectively. Vis-IV-60 has identical-sized 60 balls on a 3*4*5 grid of identical distances between adjacent vertices. Vis-V-20, Vis-VI-6, and Vis-VII-20 have balls touching reference spheres where Vis-V-20 has identical-sized 20 ball and the others have 6 or 20 poly-sized balls, respectively. They are in ‘VISUALSET’ folder of the repository.

**Fig. 1:**
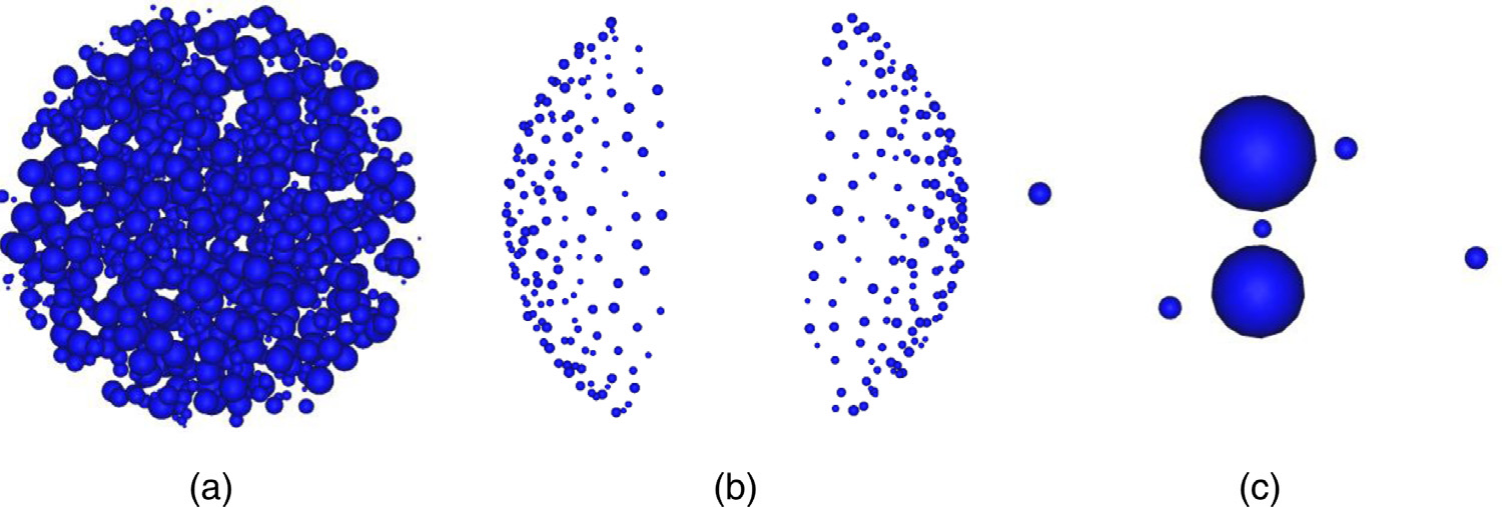
Visual examples for (a) Ball_1_^small^, (b) Ext-II-Polysized-300 and (c) ANO1

**Fig. 2:**
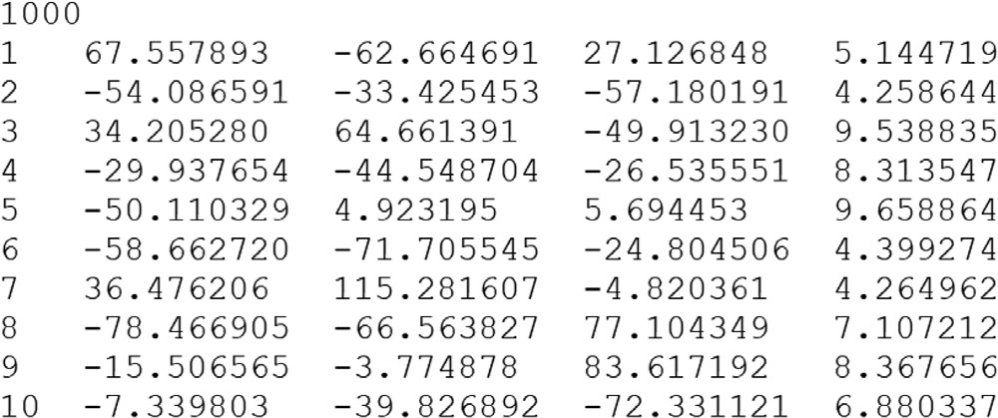
Data file format (a part of Ball_1_^small^ file from the 1^st^ to 11^th^ line). The integer of the first line denotes the number of balls in the file. Then, each following line contains the definition of each ball with 5 fields in order: id; x-, y-, z- coordinate of ball center; radius of ball.

Fig. 1 shows visual examples: (a) Ball_1_^small^, (b) Ext-II-Polysized-300, and (c) ANO1. Fig. 2 shows a part of the Ball_1_^small^ file from the 1^st^ to 11^th^ line. The integer 1000 of the first line denotes the number of balls. Each following line contains the definition of each ball with 5 fields in order: id (integer); x-, y-, z- coordinate of ball center (floating-point number); radius of ball (floating-point number). For example, a ball is defined as id: 1, center point: (67.557893, -62.664691, 27.126848), and radius: 5.144719 by the second line. The floating-point number is represented up to six decimal places. Each file in BALLCLOUD, BALLSMALLSET[1,10], EXTREMESET, ANOMALYSET, VISUALSET follows the data format.

## 2 Experimental Design, Materials and Methods

For BALLCLOUD, BALLSMALLSET[1,10], EXTREMESET, ANOMALYSET, VISUALSET, each file is created by the following generation rules. All codes are written in C++ language and run in Window 10 operating system.

1) Sets of random balls within a spherical container: BALLCLOUD, BALLSMALLSET[1,10], VISUALSET (Vis-I-10)

The radius *r_c_* of the spherical container *C* centered at origin is calculated by the Eqs. (1) and (2) where *N, r_min_, r_max_,* and ρ(=0.1) are the number of balls, the minimum / the maximum radius of balls, and expected packing ratio, respectively. This idea is a 3D version of a random disk generation rule [6]. The radius set *R* is only used to calculate the radius *r_c_* of the container. Each ball is randomly generated in an axis-aligned bounding box of the container *C.* That is, a center point (*x, y*) and radius *r* of a ball is picked from independent uniform distributions on [*-r_c_, r_c_*], [*-r_c_, r_c_*] and [*r_min_, r_max_*], respectively. If a ball is both completely in the container *C* and intersection-free from other balls, it is chosen. If not, a new randomly positioned ball with the same radius *r* is generated and tested again.
  (1) $$R=\{r_{i}\;|\;r_{min}+\left(r_{max}-r_{min}\right)*\left(\frac{i}{N+1}\right),\;i=1,2,\ldots ,N\}$$
  (2) $$\rho =\frac{\cup \left(Volume\;of\;balls\right)}{\left(Volume\;of\;container\right)}=\frac{\sum r_{i}^{3}}{r_{c}^{3}}$$

2) Sets of random balls touching a reference sphere: EXTREMESET, VISUALSET (Vis-V-20, Vis-VI-6, Vis-VII-20)

Assume that the reference sphere (*RS*) is centered at origin *O* and has radius *r_s_*. To generate a center point *P* of a ball tangent to *RS* from outside, a method to pick a random point *Q* on the surface of a unit sphere is used [7]. The center point *P* of a ball with radius *r* is generated by the Eq. (3) with the picked point *Q*. If a ball is intersection-free from other balls, it is chosen. If not, a new random ball with the same radius *r* is generated and tested again. Two consecutive chosen balls centered at *P_1_* and *P_2_* is reinforced to satisfy ∠*P_1_OP_2_* < 0.1π.
  (3) $$\vec{OP}=\left(r_{s}\;+r\right)\;\vec{OQ}$$

3) Set of balls on a grid: VISUALSET (Vis-IV-60)

Each ball is located in (-15.0 + 15.0*i, -22.5 + 15.0*j, -30.0 + 15.0*k) with radius 5.0 where 0≤i≤2,0≤j≤3,0≤k≤4.

4) Sets of balls for anomaly case in Voronoi diagram: ANOMALYSET, VISUALSET (Vis-II-5, Vis-III-5)

Each file is generated manually to represent each anomaly case.

## Ethics Statements

This work did not include work involved with human subjects, animal experiments or data collected from social media platforms.

## CRediT Author Statement

**Chanyoung Song:** Writing, Dataset maintenance; **Mokwon Lee:** Methodology, Software; **Seunghwan Choi:** Writing – reviewing, Dataset maintenance; **Deok-Soo Kim:** Supervision.

## Declaration of Competing Interest

The authors declare that they have no known competing financial interests or personal relationships that could have appeared to influence the work reported in this paper.
